# Supplementary material for: Causal Relationship between Aging and Anorexia Nervosa: A White-Matter-Microstructure-Mediated Mendelian Randomization Analysis
Source: Biomedicines. 2024 Aug 16;12(8):1874. doi: 10.3390/biomedicines12081874 (PMC11351342; doi:10.3390/biomedicines12081874)
Supplement: Supplementary file 1 [file biomedicines-12-01874-s001.zip › Supplementary Figure/Figure S8/Leaveoneout plot of MR analysis for TL to ubm-b-2044.pdf]

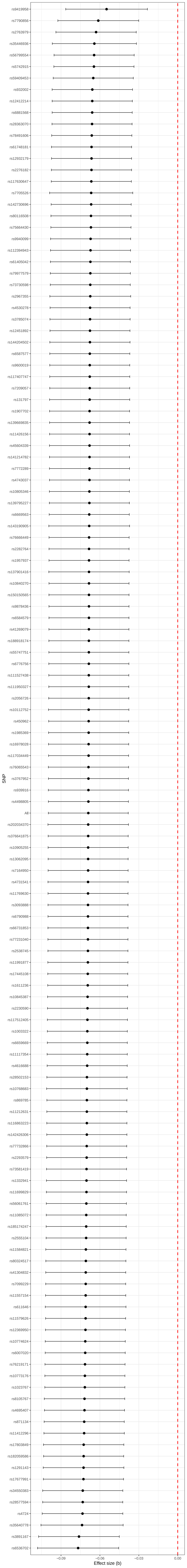
rs9419958
rs7790856
rs2763979
rs35446936
rs56799554
rs5742915
rs59409453
rs932002
rs12412214
rs6881568
rs28363070
rs78491606
rs61748181
rs12932179
rs2276182
rs117630647
rs7705526
rs142730696
rs80116508
rs75664430
rs9940099
rs112394943
rs61405042
rs79977579
rs73730598
rs2967355
rs4530278
rs3785074
rs12451892
rs144204502
rs6587577
rs9600019
rs117407747
rs7209057
rs131797
rs1907702
rs139669835
rs11426156
rs45604339
rs141214782
rs7772289
rs4743037
rs10805346
rs139795227
rs6669563
rs143190905
rs76666449
rs2282764
rs1957937
rs137901416
rs10840270
rs150150565
rs9878436
rs6584579
rs41269079
rs188918174
rs55747751
rs6776756
rs111527438
rs111950327
rs2056726
rs10112752
rs450962
rs1985369
rs16978028
rs117034449
rs76065543
rs3767952
rs939916
rs4498805
All
rs202034370
rs376641875
rs10905255
rs13062095
rs7164950
rs4731541
rs11769630
rs3093888
rs6790988
rs66731853
rs77231040
rs2538745
rs11991877
rs17445108
rs1611236
rs10845387
rs2230590
rs117512405
rs1003322
rs6659669
rs11117354
rs4616688
rs28502153
rs10768683
rs869785
rs11212631
rs116863223
rs142426306
rs77732866
rs2293579
rs73581419
rs1332941
rs11699829
rs56061761
rs11085072
rs185174247
rs2555104
rs11584821
rs80324517
rs41304832
rs7099229
rs11557154
rs611646
rs11579626
rs12369950
rs10774624
rs6007020
rs76219171
rs10773176
rs1023767
rs8105767
rs4695407
rs871134
rs11412296
rs17803849
rs182059586
rs1291143
rs17677991
rs34550383
rs28577594
rs4724
rs35640778
rs3891167
rs6536702
SNP
-0.09
-0.06
-0.03
0.00
Effect size (b)
